# Eco-friendly, stability-indicating micellar HPLC-UV method for simultaneous determination of clindamycin phosphate and adapalene in gel formulations

**DOI:** 10.1186/s13065-025-01669-x

**Published:** 2025-11-15

**Authors:** Bassant Samy, Mokhtar M. Mabrouk, Mohamed A. Abdel Hamid, Hytham M. Ahmed

**Affiliations:** 1 Pharmaceutical Analysis Department, Faculty of Pharmacy, Menoufia University, Shebin Elkom, Menoufia Egypt; 2 Department of Pharmaceutical Analytical Chemistry, Faculty of Pharmacy, Tanta University, Tanta, El Gharbeia Egypt; 3 Department of Pharmaceutical Chemistry, Faculty of Pharmacy, Alsalam University, Kafr El Zayat, El Gharbeia Egypt; 4 Pharmaceutical Analytical Chemistry Department, Faculty of Pharmacy, Menoufia National University, 70 km Cairo-Alexandria agricultural road, Tukh Tanbisha, Menoufia Egypt

**Keywords:** Clindamycin phosphate, Adapalene, Micellar liquid chromatography, GAPI

## Abstract

A rapid, accurate, sensitive, and eco-friendly micellar liquid chromatography method has been successfully developed and validated for the simultaneous determination of clindamycin and adapalene in their bulk forms and combined dosage gel formulations. The separation was performed on a BDS HYPERSIL C18 column (150 × 4.6 mm, 5 μm), employing a micellar mobile phase made up of 0.07 M sodium dodecyl sulfate, 0.3% triethylamine, 0.02 M orthophosphoric acid (pH adjusted to 3.0), and 14% isopropanol (v/v). Detection was carried out using UV at 210 nm. The method exhibited linearity in the ranges of 100–500 µg/mL for clindamycin phosphate and 10–50 µg/mL for adapalene, with detection limits of 13.4 µg/mL and 1.4 µg/mL, respectively. The developed method was effectively applied to the analysis of a laboratory-prepared co-formulated gel, yielding high recovery rates. The greenness of the method was further confirmed through assessment with the Green Analytical Procedure Index (GAPI). The method was also validated as a stability-indicating assay for clindamycin phosphate and adapalene under various stress conditions, demonstrating its robustness and applicability for routine quality control.

**Supplementary Information:**

The online version contains supplementary material available at 10.1186/s13065-025-01669-x.

## Introduction

Acne vulgaris is a prevalent skin condition that affects around 85% of adolescents, though it may develop at any stage of life. It is an inflammatory disease of the pilosebaceous units and is often associated with psychosocial distress. Recently, new therapeutic combinations of topical formulations for acne treatment have been developed and now form the backbone of effective management strategies [1–3].

**Fig. 1:**
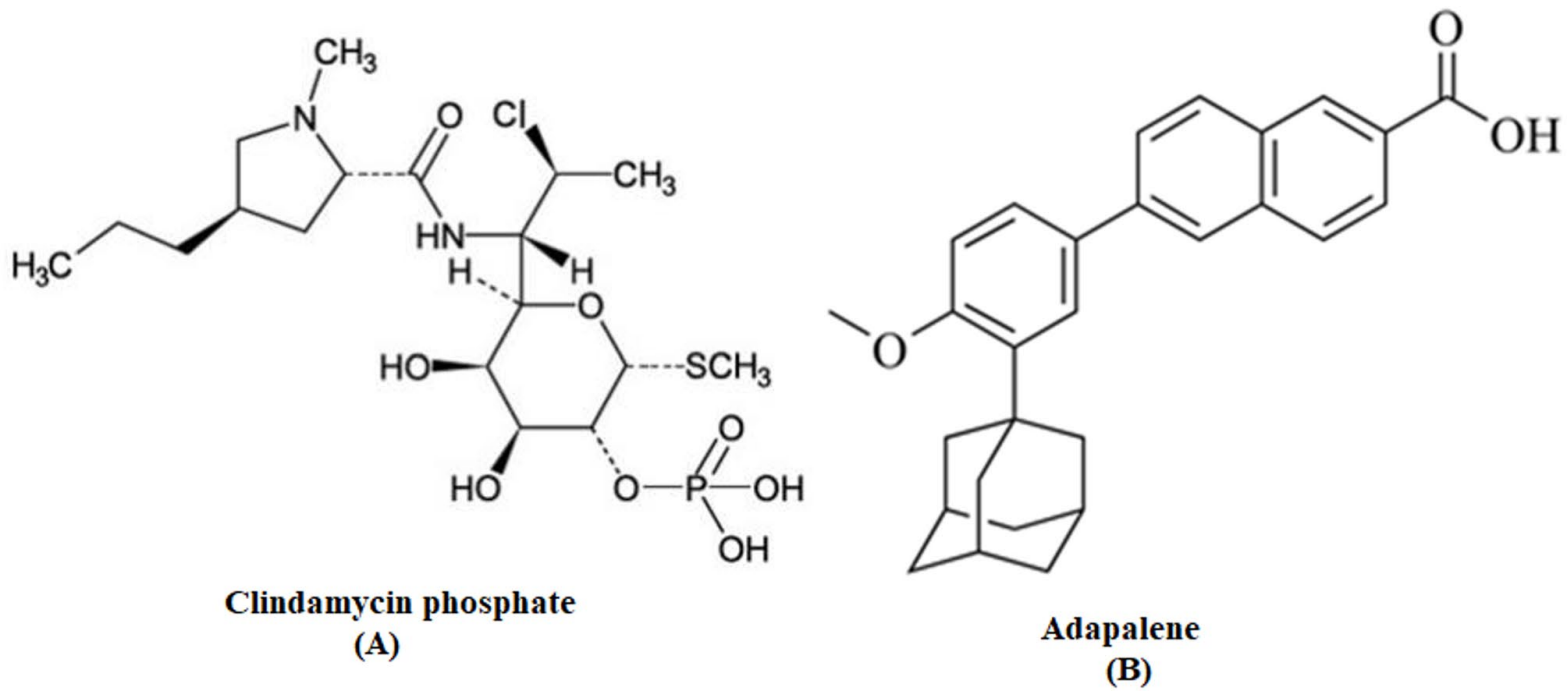
chemical structure of Clindamycin phosphate (**A**), Adapalene (**B**)

Among the various drugs available for acne treatment, clindamycin phosphate (CID) and adapalene (ADA) are frequently combined and applied topically to manage mild to moderate acne inflammation. Clindamycin phosphate (Fig. 1A) is a lincosamide antibacterial agent effective against Gram-positive aerobic bacteria as well as Gram-positive and Gram-negative anaerobes. When applied topically, CID has shown efficacy against Cutibacterium acnes [4, 5]. Adapalene (Fig. 1B) is a third-generation retinoid with improved efficacy and photostability compared to earlier generations. Retinoids possess anti-inflammatory properties and correct abnormal follicular keratinization [6, 7].

Several reversed-phase HPLC methods have been reported for the simultaneous determination of clindamycin phosphate and adapalene in topical gels [8–10]. However, these procedures typically employ large amounts of hazardous organic solvents (up to 50% acetonitrile) and/or gradient elution, which increasing cost, environmental burden, and operational complexity [11]. In contrast, micellar liquid chromatography (MLC) offers a greener and simpler alternative by using aqueous surfactant solutions with minimal organic modifier. The present work therefore introduces, optimizes, and validates a rapid, eco-friendly, isocratic MLC–UV assay requiring only 14% isopropanol. This assay demonstrates both environmental and practical advantages over existing methods and fulfilling the criteria of the Green Analytical Procedure Index (GAPI).

Micellar liquid chromatography (MLC) presents an environmentally friendly substitute for conventional reversed-phase liquid chromatography. It utilizes aqueous surfactant solutions at concentrations exceeding the critical micelle concentration, along with a minimal amount of organic solvent, to improve both elution strength and efficiency. MLC enables the separation of compounds with varied chemical properties using significantly less organic solvent and without the need for gradient elution [12–14].

This study introduces an eco-friendly and sensitive micellar liquid chromatography–UV technique for the simultaneous analysis of CID and ADA in a laboratory-prepared combined gel formulation. Unlike previously reported methods, this approach notably minimizes the use of organic solvents. The method was validated according to ICH guidelines [15], and chromatographic parameters were optimized to achieve effective separation. Additionally, the method was evaluated as a stability-indicating assay for CID and ADA under various stress conditions. Assessment of the method’s greenness was carried out using the GAPI tool [16].

## Materials and methods

### Apparatus

The chromatographic analyses were conducted utilizing an Agilent 1260 Infinity device featuring a (G1311C) quaternary pump, a thermostatted column compartment (G1316A), an autosampler (G1329B) with an injection volume range of 0.1–100 µL, and a UV detector. Data acquisition and processing were carried out using Agilent OpenLAB software. Separation was carried out on a BDS Hypersil C18 column (150 × 4.6 mm, 5 μm particle size). For the forced-degradation experiments, the prepared solutions were incubated at 30 °C in a thermostatically controlled water bath (Memmert model WNB 22, Germany) before neutralization and dilution. pH values were measured using a HANNA pH 211 m fitted with a double-junction glass electrode.

### Materials and reagents

Clindamycin phosphate (CID) and adapalene (ADA), each with a stated purity of 99.8%, were kindly provided by NODCAR, Egypt. HPLC-grade solvents, including methanol and isopropanol, were obtained from Sigma-Aldrich (Germany). Triethylamine (TEA) and orthophosphoric acid were purchased from Riedel-deHaën (Honeywell). Sodium dodecyl sulfate (SDS) and Hydrogen peroxide (30% w/v, analytical grade) was purchased from Sigma-Aldrich (USA). A 20% v/v solution was prepared in purified water for use in oxidative degradation studies. Sodium hydroxide (NaOH) pellets were obtained from GIO-Chem. Hydrochloric acid (HCl, 37%) was purchased from Fisher Chemical (UK). Excipients used in the preparation of the gel formulation, such as Carbopol 934 polymer and propylene glycol (99.9% purity), were kindly supplied by the Pharmaceutical Technology Department, Faculty of Pharmacy-Tanta University.

### Chromatographic conditions

- *Column* BDS HYPERSIL C_18_ (150 × 4.6 mm, 5 μm particle size).
- *Mobile phase* consisted of 0.07 M SDS, 0.3% TEA, 0.02 M solution of orthophosphoric acid, pH adjusted to 3.0, mixed with 14% v/v isopropanol (ratio 85% aqueous :14% isopropanol v/v). To prepare the mobile phase, the appropriate amounts of SDS and orthophosphoric acid were dissolved in water, followed by the addition of TEA and isopropanol. The pH was adjusted to 3.0 using phosphoric acid. The final solution was filtered through a 0.22 μm nylon membrane filter (Millipore, Ireland) and sonicated for 30 min.
- *Flow rate* isocratic, 1mL/min.
- *Temperature* system was operated at 40ºC.
- *Detection* UV detection at λ = 210 nm. Detection was carried out using UV at 210 nm, selected as it provided maximum sensitivity for both analytes based on their UV absorption spectra (Figure S6).
- *Injection volume* 50 μL.

The column was conditioned for 30 min before analysis.

### Stock standard solutions

Precisely weighed 25 mg of each powder of CID and ADA were each transferred into separate 25 mL volumetric flasks to prepare standard stock solutions with a concentration of 1000 µg/mL. Clindamycin was dissolved in methanol, while ADA was dissolved in tetrahydrofuran (THF). The prepared solutions were stored in a refrigerator at 4 °C and remained stable for up to one week. Tetrahydrofuran was used solely to prepare a concentrated stock solution of adapalene because the compound is practically insoluble in water, ethanol, and methanol at the required concentration. After preparation, the THF stock solution was further diluted with the micellar mobile phase before injection, minimizing the amount of THF introduced into the chromatographic system. In future work we will investigate greener alternatives (e.g., propylene glycol, dimethyl sulfoxide) for stock-solution preparation.

#### Stability of standard solutions

Stock solutions of clindamycin phosphate and adapalene were prepared freshly and stored in tightly closed amber glass vials at 4 °C. Stability studies showed no significant change in peak areas or retention times during the first 7 days, confirming that both analytes remained stable over this period. After one week, a gradual decrease in the peak area of adapalene (5–8% by day 10) was observed; for clindamycin phosphate, the decline was more moderate (3–5%). Therefore, stock solutions were not used beyond 7 days. To ensure accuracy, fresh stock solutions were prepared weekly, and all working standard solutions were prepared daily by appropriate dilution of the stock solutions with the mobile phase immediately before analysis.

### General procedure

#### Construction of calibration curves

Working standard solutions with concentration ranges of 100–500 and 10–50 µg/mL for CID and ADA, respectively, were formed by suitable diluting the stock standard solutions with the mobile phase. Under the optimized chromatographic conditions, 50 µL of each prepared solution were injected in triplicate. The regression equations were established by correlating the mean peak areas with the respective concentrations of CID and ADA using calibration plots.

#### Analysis of the laboratory prepared gel

As ADAPCLIN^®^ gel is not commercially available in Egyptian pharmacies, a laboratory-prepared gel formulation was prepared. The gel contained 1 g of CID and 0.1 g of ADA. Initially, 2% w/w Carbopol 934 was dispersed in 75 g of distilled water with continuous stirring until a uniform suspension was formed. Subsequently, 4% sodium hydroxide (NaOH) was added dropwise with constant stirring to form the gel base, and the volume was adjusted to 94 g with distilled water. Separately, the accurately weighed amounts of CID (1 g) and ADA (0.1 g) were levigated with 5 g of propylene glycol, then incorporated into the gel base with thorough mixing.

For analysis, an accurately weighed 3 g portion of the prepared gel was introduced into a 100 mL volumetric flask. It was mixed with 30 mL of tetrahydrofuran and subjected to sonication for 10 min. The solution volume was completed to 100 mL using the mobile phase, resulting in a final concentration of 300 µg/mL of CID and 30 µg/mL of ADA. Following the procedure detailed in Sect. Construction of calibration curves, the concentrations of CID and ADA were evaluated based on the appropriate regression equations.

#### Solution for forced degradation studies

All degradation samples were evaluated against a standard solution containing 100 µg/mL of CID and 10 µg/mL of ADA for comparison to see if there was a decrease in the peak area of both standard CID and ADA or the appearance of any new degradation peaks. The percentage of degradation was calculated by subtracting the CID or ADA %recovery.

I. * Acid degradation study* 1mL of CID and ADA stock solution was mixed gently with 1mL of 0.5 N hydrochloric acid and incubated for 10 min at 30 °C. The acidic mixture was then neutralised with 0.5 N sodium hydroxide to approximately pH 7. The neutralised solution was diluted to 10 mL with the micellar mobile phase to obtain final concentrations of 100 µg mL⁻¹ CID and 10 µg mL⁻¹ ADA. A 50 µL aliquot of the prepared sample was injected, and the chromatogram was recorded to assess sample stability.
II. * Alkali degradation study* 1mL of CID and ADA stock solution was mixed gently with 1mL of 0.5 N sodium hydroxide and incubated for 10 min at 30 °C. The basic mixture was then neutralised by addition of of 0.5 N hydrochloric acid to approximately pH 7 and diluted to 10 mL with the micellar mobile phase to obtain final concentrations of 100 µg mL⁻¹ CID and 10 µg mL⁻¹ ADA. A 50 µL aliquot of the prepared sample was injected and the chromatogram was recorded.
III. * Oxidation* 1 mL of 20% hydrogen peroxide (H_2_O_2_) was added separately to 1 mL of stock solution. The solutions were maintained at 30 °C for 10 min. The resulting solution was diluted to obtain 100 µg/mL and 10 µg/mL solutions for CID and ADA, and treated as outlined above.
IV. * Water hydrolysis* 1 mL of water (H_2_O) was added separately to 1 mL of stock solution. For 30 min, the solutions were kept at 30 °C. The resulting solution was diluted to obtain 100 µg/mL and 10 µg/mL solutions for CID and ADA, and treated as previously indicated.

## Results and discussion

### Method development

The mobile phase used was a green micellar mixture composed of 0.07 M SDS, 0.3% TEA, 0.02 M orthophosphoric acid at pH 3.0, and 14% v/v isopropanol. The method was optimized to ensure rapid and efficient separation of CID and ADA. The method provided distinct peak resolution for both drugs along with acceptable retention times: ADA eluted first at 4.09 min, followed by CID at 6.54 min.

In MLC, separation occurs through partitioning equilibria influenced by both hydrophobic and electrostatic interactions with the modified stationary phase containing surfactants [17]. Adapalene, being a lipophilic drug with a log P of 8.6 [18], interacts more strongly with the hydrophobic modified stationary phase compared to CID, which has a lower log P of 1 [19]. Thus, one might expect CID to elute first. However, considering the pKa values of CID (7.5) and ADA (3.9) at pH 3 [20, 21]. Clindamycin being a basic compound becomes positively charged at the working pH and is retained through electrostatic interaction with the negatively charged SDS-coated stationary phase, whereas ADA remains mostly neutral. This electrostatic retention accounts for the delayed elution of CID compared to ADA. A comparative summary of the proposed method with previously reported HPLC methods for the simultaneous determination of CID and ADA is presented in Table 1. The comparison clearly demonstrates the novelty and advantages of the developed micellar LC method. Unlike conventional methods that rely on high percentages of hazardous organic solvents like acetonitrile and tetrahydrofuran [8–10], the proposed method utilizes an eco-friendly micellar mobile phase containing only 14% isopropanol. Furthermore, the method offers a favorable balance of sensitivity, with a wide linearity range comparable to or better than existing methods, and efficient separation with reasonable retention times, avoiding the excessively long run times associated with some procedures.

**Table 1:** Comparison of the proposed MLC method with reported HPLC methods for the simultaneous determination of clindamycin phosphate (CID) and adapalene (ADA)

Mobile Phase Composition	Sensitivity (Linearity Range)	Retention Time (min)	Key Characteristics	Ref.
Acetonitrile: phosphate buffer pH 3.0 (60:40, v/v)	100–500 µg/mL (CID) 10–50 µg/mL (ADA)	CID: 3.03 ADA: 4.92	Isocratic. High ACN content.	[8]
Ammonium hydrogen phosphate buffer (pH 2.5):ACN: THF	20–150 µg/mL (CID) 0.5–150 µg/mL (ADA)	CID: 7.58 ADA: 11.2	Gradient elution.	[9]
0.1% OPA: ACN: THF (65:35, v/v)	100–300 µg/mL (CID) 10–30 µg/mL (ADA)	CID: 4.49 ADA: 19.1	Isocratic. Very long retention time for ADA.	[10]
0.07 M SDS, 0.3% TEA, 0.02 M orthophosphoric acid (pH 3.0), 14% (v/v) isopropanol .	100–500 µg/mL (CID) 10–50 µg/mL (ADA)	CID: 6.55 ADA: 4.10	Isocratic. Micellar LC. Eco-friendly (low isopropanol). No ACN	This work

### Method optimization

Various factors affecting the separation of ADA and CID were thoroughly investigated and incorporated into the method. These parameters include flow rate, pH, organic modifier concentration, and surfactant concentration. The goal of the investigation was to find a chromatographic system that could be operated with the shortest run time while having the best resolution and largest theoretical plate number.

#### The effect of pH

Changes in the mobile phase pH can easily cause changes in retention time, resolution, and number of theoretical plates (NTP) [22, 23]. Thus, the effect of pH was investigated in the 2.5–5.0 range (Figure S1). The NTP increased with decreasing pH for both CID and ADA, achieving its maximum at (pH 3.0) then declining to (pH 2.5). Consequently, it was decided that the ideal pH was 3.0.

#### Effect of flow rate

The flow rate effect was investigated in order to improve chromatographic efficiency and peak resolution [24]. The flow rate was varied between 0.5 and 1.5 mL/min. It was found that 1 mL/min was the ideal flow rate, which produced greater separation while maintaining a suitable retention time (Figure S2). Greater flow rates than 1 mL/min led to a reduction in the resolution between the two drugs, whereas flow rates less than 1 mL/min produced a longer retention time with lower resolution.

#### Effect of SDS concentration

The effect of changing SDS concentrations from 0.05 to 0.09 M on the number of theoretical plates (NTP) and resolution was investigated as in Figure S3. NTP and resolution for both drugs increased with increasing SDS concentration up to 0.07 M, after which they began to decrease. As a result, 0.07 M SDS was chosen as the optimum.

#### Effect of organic modifier concentration

The concentration of isopropanol was studied from 10% to 15% according to the theoretical plate number (NTP) and resolution (Figure S4). The ideal organic modifier concentration was 14%, which provided the best NTP and resolution.

As a result of the previous studies, the optimum mobile phase consisted of 0.07 M SDS, 0.3% TEA, 0.02 M solution of orthophosphoric acid, pH adjusted to 3.0, and isopropanol (85% aqueous phase and 14% isopropanol v/v).

### Method validation

The method was validated in terms of accuracy, precision, selectivity and specificity, limit of detection (LOD), limit of quantitation (LOQ), and robustness in accordance with ICH guidelines [15, 30].

#### Range of linearity

The linearity of the proposed method was established by graphing peak area against concentration (µg/mL) for each drug. The calibration curves demonstrated linearity responses over the ranges of 100–500 µg/mL for CID and 10–50 µg/mL for ADA. Statistical analysis of the data revealed strong correlation coefficients and low relative standard deviations, as presented in Table 2.

**Table 2:** Regression and quantitative parameters for the determination of clindamycin and adapalene by the proposed micellar HPLC method

Parameter	Clindamycin	Adapalene
Linearity Range (µg/mL)	100–500	10–50
Intercept (a)	-117.74	43.09
SD of Intercept (S_a_)	32.22 ^a^	21.66 ^a^
Slope (b)	7.886	48.368
SD of Slope (S_b_)	0.097 ^a^	0.653 ^a^
Coefficient of determination (r^2^)	0.9996	0.9995
SD of residual (S_y/x_)	30.72 ^a^	20.65 ^a^
Limit of detection (µg/mL)	13.4	1.4
Limit of quantitation (µg/mL)	40.8	4.4
Accuracy (mean %recovery ±%RSD)	99.95 ± 1.3 ^a^	99.6 ± 0.3 ^a^

^a^standard deviation (*n* = 3)

#### Limits of detection (LOD) and quantitation (LOQ)

The following equations were used to calculate the LOD and LOQ: LOD = 3.3 σ / S; LOQ = 10 σ / S, where S represents the slope and σ is the standard deviation of the intercept from the calibration curve’s regression line. The LOD and LOQ values for CID and ADA are summarized in Table 2.

#### Accuracy

The accuracy of the suggested approach was assessed using triplicate analysis of CID and ADA mixtures created in the laboratory within the linearity range. The proposed method recovery was found to be 99.95 ± 1.3% for CID and 99.6 ± 0.3% for ADA, demonstrating good accuracy as presented in Table 3.

**Table 3:** Accuracy of the proposed micellar HPLC–UV method for the determination of CID and ADA in laboratory-prepared mixtures within the linearity range. Values are mean recovery ± RSD (n = 3)

	Concentration taken(µg/mL)	Concentration taken(µg/mL)			Mean concentration Found(µg/mL)	%Recovery	RSD
Clindamycin	150	148.8	149.11	151.02	149.643	99.762	0.981
	250	250.8	247.6	251.2	249.866	99.946	1.611
	350	353.76	350.9	349.9	351.52	100.434	1.635
	450	448.7	447.8	450.2	448.9	99.755	0.989
Adapalene	15	14.8	14.9	15.1	14.933	99.555	0.124
	25	25.2	24.8	24.6	24.866	99.466	0.249
	35	34.9	34.7	34.8	34.8	99.428	0.081
	45	44.7	45.7	44.7	45.033	100.074	0.471

#### Precision

To assess precision, both repeatability and intermediate precision were evaluated. For repeatability, three different binary mixtures of CID and ADA were analyzed in triplicate within the same day. Intermediate precision was determined by analyzing the same mixtures over three consecutive days. The percentage relative standard deviation (%RSD) values confirm the precision of the method, as presented in Table S1.

#### Robustness

Robustness was assessed in relation to intentional small changes in conditions such as pH, the proportion of isopropanol, and flow rate. The variations in the aforementioned parameters showed no significant impact, confirming the robustness of the method. Robustness was evaluated by deliberate variation of pH (2.8 and 3.2), flow rate (0.9 and 1.1 mL min⁻¹), and isopropanol content (13.5 and 14.5% v/v), while keeping all other chromatographic conditions constant. Three replicate injections were performed under each condition (Table S2).

#### Specificity and selectivity

**Fig. 2:**
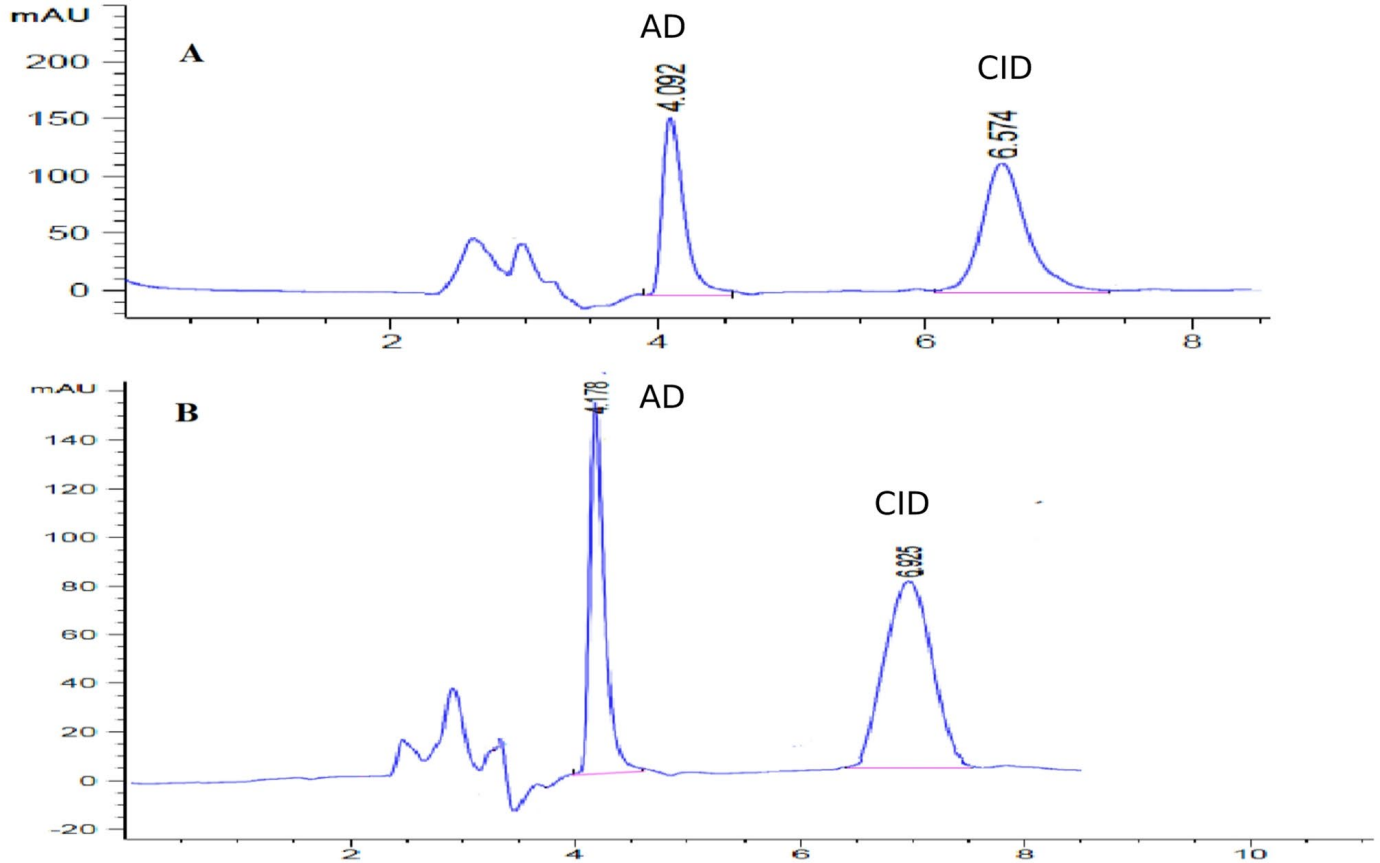
Typical chromatograms under the proposed micellar liquid chromatography conditions (BDS with flow rate 1 mL/min, detection at 210 nm, injection volume 50 µL: **A** standard mixture of containing 150 µg/mL CID and 15 µg/mL ADA. **B** laboratory-prepared gel formulation containing 300 µg/mL CID and 30 µg/mL ADA. Peak 1: ADA; Peak 2: CID

The selectivity of the proposed method was confirmed by the clear separation between the peaks of the two drugs, indicating its capability to accurately quantify each compound in the presence of the other without mutual interference (Fig. 2A). Specificity was evaluated by analyzing CID and ADA in their laboratory-prepared combined gel formulation, showing no interference from common excipients or additives, as illustrated in Fig. 2B.

### System suitability test parameters

Following the optimization of all experimental conditions affecting the chromatographic separation, the final chromatographic setup was evaluated for system suitability parameters, including resolution (R), number of theoretical plates (NTP), and symmetry factor. As shown in Table 4, the obtained results met the criteria for an acceptable analytical method.

**Table 4:** Results for system suitability tests for the developed HPLC method

Parameter	Adapalene	Clindamycin	Reference value [29]
Retention time (min)	4.097	6.549	
Resolution (Rs)	2.46	6.15	> 2
Number of theoretical plates (NTP)	4529	2245	> 2000
Symmetry	0.56	0.82	≤ 2

### Application for the determination of CID and ADA in their combined dosage form

The proposed method was used to analyse the target drugs in their laboratory synthetic co-formulated gel (Fig. 2B). The proposed method results were compared to those of reference methods [25, 26] as shown in Table 5. No significant difference was observed between the proposed method and the reference method.

**Table 5:** Application of the proposed method and comparison method for the determination of the studied drugs in their laboratory prepared mixture with excipients

Proposed method				Comparison method [25, 26]	
Conc. taken (µg/mL)		%Recovery		%Recovery	
		CID	ADA	CID	ADA
300		99.54	99.5	99.1	99.8
	30	99.1	99.06	98.8	99.03
		99.71	99.3	100.3	99.7
			100.07		100.2
Mean % Recovery ± SD		99.45 ± 0.25	99.48 ± 0.37	99.4 ± 0.64	99.6 ± 0.42
t- test		0.101	0.66		
F-test		6.3	1.4		

t- and F-test values were calculated at *p* = 0.05

### Results of forced degradation studies

To confirm that the proposed micellar liquid chromatographic (MLC) method is stability-indicating, clindamycin phosphate (CID) and adapalene (ADA) were subjected to deliberate stress conditions recommended by ICH: acidic (0.5 N HCl, 10 min, 30 °C), alkaline (0.5 N NaOH, 10 min, 30 °C), oxidative (20% H₂O₂, 10 min, 30 °C) and water stress (30 min, 30 °C). After neutralisation and dilution with the mobile phase the samples were analysed under the same chromatographic conditions as the unstressed controls.

**Fig. 3:**
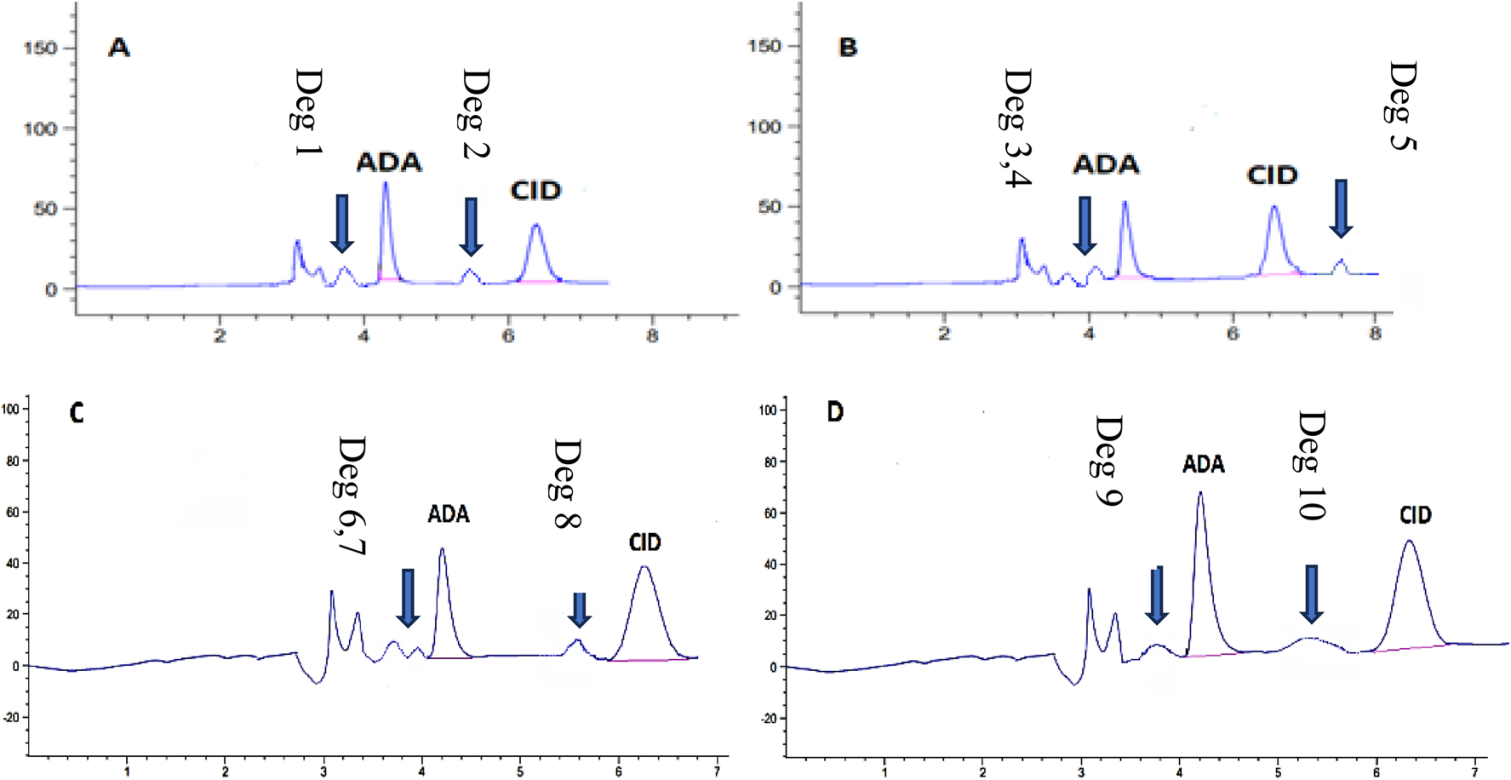
Chromatograms of clindamycin phosphate (CID) and adapalene (ADA) under forced-degradation conditions: **A** acidic (0.5 N HCl, 10 min, 30 °C); **B** alkaline (0.5 N NaOH, 10 min, 30 °C); (C) oxidative (20% H₂O₂, 10 min, 30 °C); **D** water stress (30 min, 30 °C). Parent peaks for ADA (RT ≈ 4.09 min) and CID (RT ≈ 6.50 min) are labeled. Newly formed peaks correspond to degradation products; their retention times and relative areas are listed in Table 6

In all cases the parent peaks of ADA (RT ≈ 4.09 min) and CID (RT ≈ 6.50 min) remained sharp and baseline-resolved. Figure 3(A–D) shows the chromatograms obtained under each stress condition with new peaks annotated as “Deg-1”, “Deg-2” and their retention times. Table 6 summarises the percentage recovery, calculated degradation and retention times of these newly formed peaks.

**Table 6:** Percentage recovery and calculated degradation of clindamycin phosphate (CID) and adapalene (ADA) under various stress conditions with retention times (RT) and relative areas of newly formed peaks observed in Fig. 3A–D

Degradation content	Clindamycin			Adapalene			New peaks (RT min; relative to parent %)
	Concentration added	%Recovery	%Degradation	Concentration added	%Recovery	%Degradation	
Acid (0.5 N 10 min 30 °C)	100	89.020	10.97	10	89.0	10.925	ADA: 3.75 min (10%) CID: 5.50 min (10%)
Oxidation (20%H2O2 10 min 30 °C)		90.419	9.580		86.5	13.489	ADA: 3.70 min (6%), 3.91 min (7%) CID: 5.59 min (9%)
Alkali (0.5 N 10 min 30 °C)		91.8	8.2		85.6	14.395	ADA: 3.69 min (8%), 4.12 min (6%) CID: 7.51 min (8%)
Water (30 min 30 °C)		94.22	5.777		92.86	7.137	ADA: 3.68 min (7%) CID: 5.20 min (5%)

Under acidic stress (Fig. 3A) the recovery of CID and ADA decreased to about 89% (≈ 11% degradation). New peaks appeared at RT 3.75 min for ADA and 5.50 min for CID, each representing about 10% of the parent peak area. These changes are consistent with acid-catalysed hydrolysis of the CID and possible isomerisation/protonation of ADA’s retinoid moiety.

Under alkaline stress (Fig. 3B) CID recovered 91.8% (≈ 8% degradation) and ADA 85.6% (≈ 14% degradation), with peaks at RT 3.69 min (8%) and 4.12 min (6%) for ADA and 7.51 min (8%) for CID. This behaviour is suggestive of base-catalysed cleavage of phosphate and sugar-like moieties in CID and double-bond isomerisation of ADA.

Under oxidative stress (Fig. 3C) CID showed 90.4% recovery (≈ 9.6% degradation) and ADA 86.5% recovery (≈ 13.5% degradation), with new peaks at RT 3.70 min (6%) and 3.91 min (7%) for ADA and 5.59 min (9%) for CID. Clindamycin contains a thioether and tertiary amine which can form sulfoxide or N-oxide derivatives; ADA’s polyaromatic system may undergo hydroxylation or peroxidation, all leading to earlier-eluting, more polar products.

Under water stress (Fig. 3D) only small changes were seen (≈ 5–7% degradation) with minor additional peaks at RT 3.68 min for ADA and 5.20 min for CID, indicating limited hydrolysis or isomerisation under neutral conditions.

Although full structural elucidation of the degradation products was not attempted, the consistent decrease of the parent peaks accompanied by appearance of separate degradant peaks under all stress conditions demonstrates that the proposed MLC method cleanly resolves CID and ADA from their degradation products and is therefore stability-indicating.

### Assessment of the greenness of the proposed method

**Fig. 4:**
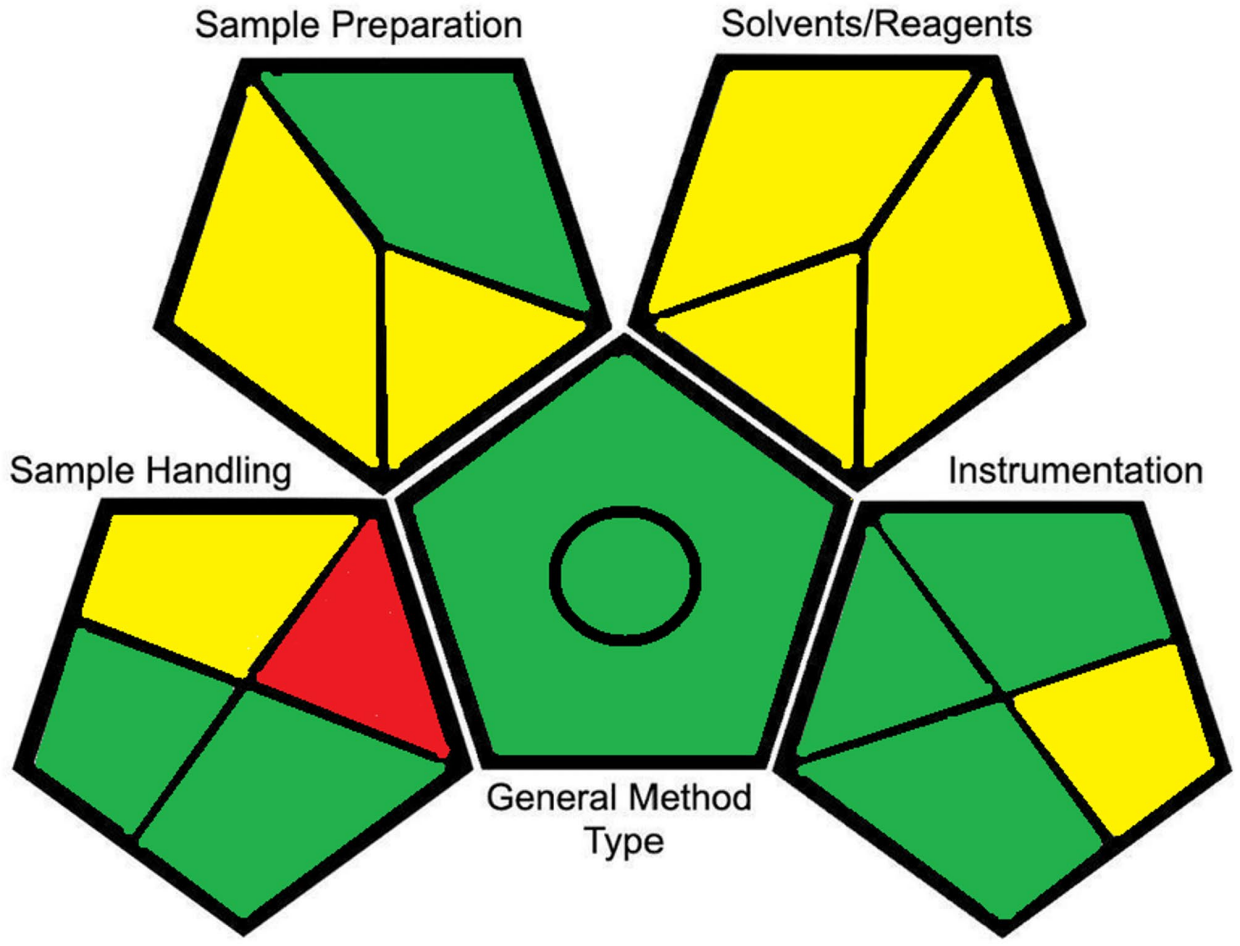
The green assessment profile for the proposed HPLC-UV method using the GAPI tool

Green analytical methods are designed to function with little to no use of reagents, use less energy, and produce no waste. However, eliminating the use of reagents is not possible in many analytical processes, hence it’s highly challenging to replace hazardous reagents with safer, easily degradable alternatives, eliminate or reduce the risk of wastes, and minimize the number of steps in a particular analytical process [16, 27, 28]. GAPI is a relatively new tool for evaluating an analytical procedure’s greenness [16]. It consists of five pentagrams that assess the environmental impact of each step of the proposed method using a color-coded scheme: red for high impact, yellow for moderate impact, and green for low impact. The proposed MLC method exhibited excellent eco-friendly attributes, confirming its suitability as a green HPLC technique (Fig. 4). In addition to the Green Analytical Procedure Index (GAPI), the greenness of the proposed method was assessed using the Analytical Eco-Scale and AGREE. The AGREE evaluation (Figure S6) produced a composite score of **0.79** and as shown in the color-coded wheel, with most criteria in the green zone, indicating a highly green analytical procedure. Individual segments reflect favorable performance in miniaturization, waste generation, safety of reagents, and sample throughput.

The Analytical Eco-Scale, which penalizes factors such as hazardous reagents, energy consumption and waste, gave a score of **~ 80**. According to the Eco-Scale classification, a score > 75 represents an “excellent green” method.(Table S3 summarizes the calculation).

These complementary evaluations show that, despite the limited use of THF to dissolve adapalene at the stock-solution stage, the overall method remains environmentally friendly due to the small injection volume (50 µL), micellar mobile phase, and low solvent consumption per analysis. Taken together, GAPI, AGREE and Eco-Scale confirm the high environmental compatibility of the proposed method.

## Concluding remarks

In this work we have developed and validated, for the first time, a simple, precise and accurate micellar liquid chromatography (MLC) method for the simultaneous determination of clindamycin phosphate (CID) and adapalene (ADA) in their combined gel formulation.

Unlike previously reported RP-HPLC methods, the proposed procedure employs a micellar mobile phase containing only 14% isopropanol and no acetonitrile or other Class-2 solvents, thereby markedly reducing hazardous solvent consumption and aligning with green-analytical-chemistry principles.

The method shows excellent linearity (100–500 µg/mL for CID and 10–50 µg/mL for ADA) with low detection limits (13.4 µg/mL and 1.4 µg/mL, respectively) and good precision and accuracy, making it highly suitable for routine quality-control laboratories.

Comprehensive forced-degradation studies under ICH-recommended stress conditions demonstrated the stability-indicating power of the assay, with degradant peaks clearly separated from the parent drugs.

Combined evaluation with GAPI, AGREE and the Analytical Eco-Scale confirms that the method is an “excellent green” analytical approach. These attributes green chemistry compliance, simple isocratic operation, rapid run time, and stability-indicating capability constitute the novelty and importance of this work compared with previously published methods.

## Supplementary Information

Below is the link to the electronic supplementary material.

Supplementary Material 1

## Data Availability

The data sets used and analyzed are available from the corresponding author upon reasonable request.

## Abbreviations

ADA: Adapalene
CID: Clindamycin
GAPI: Green analytical procedure index
MLC: Micellar liquid chromatography
NTP: Number of theoretical plate
SDS: Sodium dodecyl sulphate
TEA: Triethylamine

## References

[1] LAYTON, Alison M, RAVENSCROFT, Jane. Adolescent acne vulgaris: current and emerging treatments. Lancet Child Adolesc Health. 2023;7(2):136–44. 36525984 10.1016/S2352-4642(22)00314-5

[2] Shah J, Parmar D. A complete review on acne vulgaris. J Adv Med Dent Sci Res. 2015;3:20.

[3] Dawn EICHENFIELD, SPRAGUE Z, Jessica EICHENFIELD, Lawrence F. Management of acne vulgaris: a review. JAMA. 2021;326(20):2055–67. 34812859 10.1001/jama.2021.17633

[4] LUCHIAN, Ionut, et al. Clindamycin as an alternative option in optimizing periodontal therapy. Antibiotics. 2021;10(7):814. 34356735 10.3390/antibiotics10070814 PMC8300806

[5] YANG, Yingying, et al. Recent development and fighting strategies for Lincosamide antibiotic resistance. Clin Microbiol Rev. 2024;37(2):e00161–23. 38634634 10.1128/cmr.00161-23 PMC11237733

[6] Rusu A, Tanase C, Pascu G-A, Todoran N. Recent advances regarding the therapeutic potential of adapalene. Pharmaceuticals. 2020;13:217. 32872149 10.3390/ph13090217 PMC7558148

[7] CHEN, Wangqing, et al. Retinoids as an Immunity-modulator in dermatology disorders. Arch Immunol Ther Exp. 2019;67(6):355–65. 10.1007/s00005-019-00562-5 31552446

[8] Khatri RH, Patel RB, Patel MR. A new RP-HPLC method for Estimation of clindamycin and adapalene in gel formulation: development and validation consideration. TJPS. 2014;38:44–8.

[9] Modi PB, Shah NJ. Novel stability-indicating RP-HPLC method for the simultaneous Estimation of clindamycin phosphate and adapalene along with preservatives in topical gel formulations. Sci Pharm. 2014;82:799–814. 26171325 10.3797/scipharm.1404-01 PMC4475806

[10] Navkhare M, Gaidhane H, Chaple D, Ingale P, Ghodekar S. Validated stability indicating analytical method for the determination of clindamycin phosphate and adapalene in topical formulation. Anal Chem. 2013;13:e5.

[11] YABRÉ, Moussa, et al. Greening reversed-phase liquid chromatography methods using alternative solvents for pharmaceutical analysis. Molecules. 2018;23(5):1065. 29724076 10.3390/molecules23051065 PMC6100308

[12] Esteve-Romero J, Albiol-Chiva J, Peris-Vicente J. A review on development of analytical methods to determine monitorable drugs in serum and urine by micellar liquid chromatography using direct injection. Anal Chim Acta. 2016;926:1–16. 27216388 10.1016/j.aca.2016.04.026

[13] Stępnik KE. A concise review of applications of micellar liquid chromatography to study biologically active compounds. Biomed Chromatogr. 2017;31:e3741. 10.1002/bmc.3741 27076037

[14] El-Shaheny RN, El-Maghrabey MH, Belal FF. Micellar liquid chromatography from green analysis perspective. Open Chem. 2015, 13.

[15] Swartz ME, Krull IS, editors. Analytical method development and validation. 1st ed. Boca Raton, FL: CRC; 1997. 10.1201/9781315275161.

[16] Płotka-Wasylka J. A new tool for the evaluation of the analytical procedure: green analytical procedure index. Talanta. 2018;181:204–9. 29426502 10.1016/j.talanta.2018.01.013

[17] WARD, Timothy J, WARD, Karen D. Solubilization in micellar separations. Solubilization in surfactant aggregates. CRC; 2020. pp. 517–40.

[18] Pereira RL, Leites FI, Paese K, Sponchiado RM, Michalowski CB, Guterres SS, Schapoval EE. Hydrogel containing adapalene-and dapsone-loaded lipid-core nanocapsules for cutaneous application: development, characterization, in vitro irritation and permeation studies. Drug Dev Ind Pharm. 2016;42:2001–8. 27161601 10.1080/03639045.2016.1188110

[19] Li Z, Zhang Y, Lin M, Ouyang P, Ge J, Liu Z. Lipase-catalyzed one-step and regioselective synthesis of clindamycin palmitate. Org Process Res Dev. 2013;17:1179–82.

[20] Pavlović N, Bogićević IA, Zaklan D, Đanić M, Goločorbin-Kon S, Al-Salami H, Mikov M. Influence of bile acids in hydrogel pharmaceutical formulations on dissolution rate and permeation of clindamycin hydrochloride. Gels. 2022;8:35. 35049570 10.3390/gels8010035 PMC8774652

[21] Gökçe BB, Boran T, Emlik Çalık F, Özhan G, Sanyal R, Güngör S. Dermal delivery and follicular targeting of adapalene using PAMAM dendrimers. Drug Delivery Translational Res. 2021;11:626–46. 10.1007/s13346-021-00933-6 33666878

[22] GANESH V, et al. Retention behaviour of analytes in reversed-phase high‐performance liquid chromatography—A review. Biomed Chromatogr. 2023;37(7):e5482. 35962484 10.1002/bmc.5482

[23] HEERING, Agnes, et al. Improved pH measurement of mobile phases in reversed-phase liquid chromatography. Analyst. 2024;149(5):1481–8. 38314857 10.1039/d3an02029k

[24] McCalley DV. Study of the selectivity, retention mechanisms and performance of alternative silica-based stationary phases for separation of ionised solutes in hydrophilic interaction chromatography. J Chromatogr A. 2010;1217:3408–17. 20362994 10.1016/j.chroma.2010.03.011

[25] Ibrahim F, El-Deen AK, Abass E, Shimizu SA. An ecofriendly green liquid chromatographic method for simultaneous determination of nicotinamide and clindamycin phosphate in pharmaceutical gel for acne treatment. J Food Drug Anal. 2017;25:741–7. 28911660 10.1016/j.jfda.2016.09.009 PMC9328823

[26] Tolba M, Elgamal R. Determination of adapalene in gel formulation by conventional and derivative synchronous fluorimetric approaches. Application to stability studies and in vitro diffusion test. Chem Cent J. 2016, 10. 10.1186/s13065-016-0181-0 PMC4884421 27239224

[27] KUMAR¹ N, Narendra; ARCHANA MA. Comprehensive Review of Green Metric Tools for Sustainable Analytical Method Development. In: Proceedings of the International Conference on Bio-Based Environment for Sustainable Territory (IC-BEST 2024). Springer Nature, 2025. p. 132.

[28] Tobiszewski M, Marć M, Gałuszka A, Namieśnik J. Green chemistry metrics with special reference to green analytical chemistry. Molecules. 2015;20:10928–46. 26076112 10.3390/molecules200610928 PMC6272361

[29] USP USP. 791 > pH, USP 43-NF 38. United States pharmacopeial convention. Inc.; 2020. p. 7022.

[30] Pippalla S, Kumar V, Nekkalapudi AR, et al. A Novel, Stability-Indicating RP-HPLC method for simultaneous Estimation of assay and organic impurities of pyridostigmine bromide and assay of sodium benzoate in liquid oral formulation. Pharm Chem J. 2024;58:1339–47. 10.1007/s11094-024-03279-8.

