# Supplementary material for: Eco-friendly, stability-indicating micellar HPLC-UV method for simultaneous determination of clindamycin phosphate and adapalene in gel formulations
Source: BMC Chem. 2025 Nov 15;19(1):307. doi: 10.1186/s13065-025-01669-x (PMC12619446; doi:10.1186/s13065-025-01669-x)
Supplement: Supplementary file 1 — Supplementary Material 1 [file 13065_2025_1669_MOESM1_ESM.docx]

**Eco-Friendly, Stability-Indicating Micellar HPLC-UV Method for Simultaneous Determination of Clindamycin Phosphate and Adapalene in Gel Formulations**

**Bassant Samy ^1^ , Mokhtar M. Mabrouk ^2,3^, Mohamed A. Abdel Hamid ^2,3^, Hytham M. Ahmed ^1,4^**

^1^Pharmaceutical Analysis Department, Faculty of Pharmacy, Menoufia University, Shebin Elkom, Menoufia, Egypt.

^2^Department of Pharmaceutical Analytical Chemistry, Faculty of Pharmacy, Tanta University, Tanta, El Gharbeia, Egypt.

^3^Department of Pharmaceutical Chemistry, Faculty of Pharmacy, Alsalam University, Kafr El Zayat, El Gharbeia, Egypt.

^4^Pharmaceutical Analytical Chemistry Department, Faculty of Pharmacy, Menoufia National University, 70 km Cairo-Alexandria agricultural road, Menoufia, Egypt.

Corresponding author: Tel: 00201004844589

E-mail: [hmaahmed@yahoo.co.uk](mailto:hmaahmed@yahoo.co.uk)

A

**Figure S1** **Effect of pH on the chromatographic response for: (A) CID and (B) ADA**.

B

A

**Figure S2** **Effect of flow rate on chromatographic response for: (A) CID and (B) ADA.**


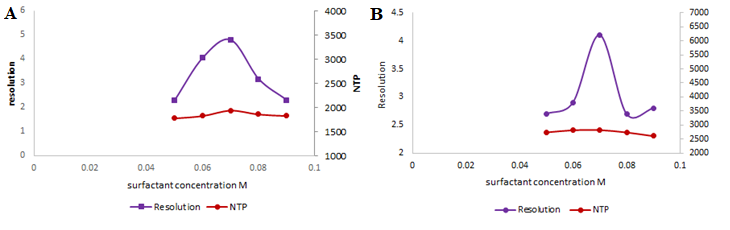


**Figure S3** **Effect of SDS concentration on chromatographic response for :(A) CID and (B) ADA.**

**Figure S4** **Effect of organic modifier concentration on chromatographic response for :(A) CID and (B) ADA.**


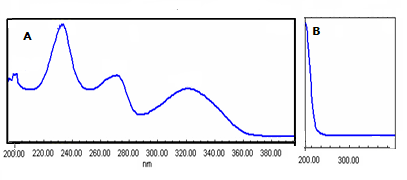


**Figure S5 UV absorption spectra of adapalene (A) and clindamycin phosphate (B) in the proposed micellar mobile phase showing strong absorbance at 210 nm, the selected detection wavelength for the chromatographic method.**

**
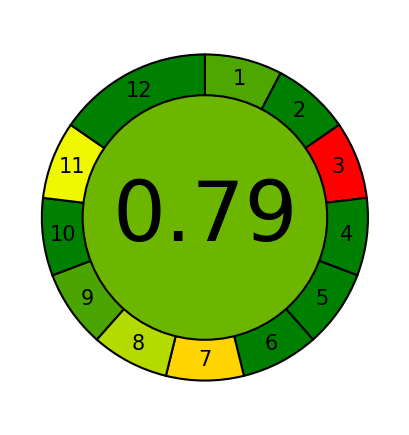
**

**Figure S6** **AGREE evaluation of the proposed micellar HPLC–UV method for clindamycin phosphate and adapalene, showing a composite greenness score of 0.79**

**Table S1**: **Intra-day and inter-day precision for the determination of CID and ADA by the proposed micellar HPLC–UV method at three concentration levels. Values are mean found concentration ± %RSD (n = 3).**

| **Precision** | **Conc. taken of CID (μg/mL)** | **Mean conc. found of CID (μg/mL) ±%RSD** | **Conc. taken of ADA (μg/mL)** | **Mean conc. Found of ADA (μg/mL) ± %RSD** |
| --- | --- | --- | --- | --- |
| Intra-day | 150 | 149.6 ± 0.98 | 15 | 14.9 ± 0.12 |
|  | 250 | 249.8 ± 1.6 | 25 | 24.9 ± 0.2 |
|  | 350 | 351.5 ± 1.6 | 35 | 34.8 ± 0.1 |
|  | | | | |
| Inter-day | 150 | 149.6 ± 0.9 | 15 | 15.1 ± 0.08 |
|  | 250 | 249.8 ± 1.1 | 25 | 25.2 ± 0.02 |
|  | 350 | 349.9 ± 1.5 | 35 | 34.9 ± 0.11 |

**Table S2**: **Robustness of the proposed micellar HPLC–UV method for the determination of CID and ADA at 350 µg/mL and 35 µg/mL, respectively, under small deliberate changes in pH, flow rate, and isopropanol content. Values are mean found concentration ± %RSD (n = 3), with corresponding retention times (Rt) ± SD and resolution (Rs).**

|  | **CID mean conc. found (μg/mL) ±%RSD** | **ADA mean conc. found (μg/mL) ± %RSD** | **CID Rt (min) ± SD** | **ADA Rt (min) ± SD** | **Resolution (Rs)** |
| --- | --- | --- | --- | --- | --- |
| **Optimum condition at PH 3 flow rate 1 isopropanol 14%** | 349.9 ± 1.5 | 34.9 ±0.11 | 6.549 ± 0.01 | 4.097 ± 0.008 | 6.15 |
| **PH (2.8)** | 347.9 ± 0.12 | 34.75 ± 0.1 | 6.545 ± 0.02 | 4.116 ± 0.005 | 6.13 |
| **PH (3.2)** | 350.5 ± 0.69 | 35.2 ± 0.13 | 6.556 ± 0.009 | 4.096 ± 0.01 | 6.11 |
| **flow rate = 1.1** | 351 ± 0.9 | 34.8 ± 0.11 | 6.547 ± 0.014 | 4.115 ± 0.026 | 6.13 |
| **flow rate = 0.9** | 351.4 ± 0.5 | 34.91 ± 0.12 | 6.555 ± 0.018 | 4.094 ± 0.009 | 6.20 |
| **Iso Propanol 13.5%** | 350.45 ± 0.65 | 34.82 ± 0.08 | 6.527 ± 0.015 | 4.11± 0.01 | 6.12 |
| **Iso Propanol 14.5%** | 348.9 ± 1.2 | 35.12 ± 0.03 | 6.51 ± 0.02 | 4.099 ± 0.005 | 6.05 |

***N.B.***: * n = 3.

**Table S3 Analytical Eco-Scale evaluation of the proposed MLC method for clindamycin phosphate and adapalene. A score of ~80 indicates an “excellent green” procedure.**

| **Factor** | **Penalty points** |
| --- | --- |
| Isopropanol (14 %) | 4 |
| Triethylamine (0.3 %) | 4 |
| Orthophosphoric acid (0.02 M) | 1 |
| SDS surfactant | 0 |
| Tetrahydrofuran (used only for ADA stock, >100-fold diluted before injection) | 6 |
| Energy use (HPLC) | 1 |
| Waste per run <10 mL | 0 |
| Total penalty points | 16 |
| **Eco-Scale score** = 100 – 16 = **84 (excellent green analysis)** | |
